# A retrospective analysis of voluntary adverse event reports to the TGA with higher THC medicinal cannabis products

**DOI:** 10.1177/00048674261445267

**Published:** 2026-05-03

**Authors:** Myfanwy Graham, Calvert Tisdale, Suzanne Nielsen

**Affiliations:** Monash Addiction Research Centre, Monash University, Melbourne, VIC, Australia

## Introduction

Since 2021, Australia has seen substantial shifts in prescribing application approvals of medicinal cannabis products, from balanced or cannabidiol (CBD)-dominant oral oil-based products to inhaled higher delta-9-tetrahydrocannabinol (THC) concentration products. In parallel, case reports of psychosis following the prescription of higher THC medicinal cannabis products have been published (Lupke et al., 2024).

Medicinal cannabis categories are defined by the Therapeutic Goods Administration (TGA) as proportional definitions between CBD and other cannabinoids, including THC. These categories (introduced in late 2021) include Category 1, CBD product (CBD > 98%), Category 2, CBD-dominant product (CBD > 60% and < 98%), Category 3, balanced product (CBD > 40% and < 60%), Category 4, THC/other cannabinoid-dominant product (CBD > 2% and < 40%) and Category 5, THC/other cannabinoid product (CBD < 2%). Almost all Category 5 products contain predominantly THC, although represent a wide range of THC concentrations, from 13% to >88% THC (Graham et al., 2026; Therapeutic Goods Administration, 2025a). There are more than 1000 unregistered medicinal cannabis products available for prescription in Australia that have not been assessed by the TGA for efficacy, safety or quality.

This study aims to describe voluntary adverse event reports involving medicinal cannabis to Australia’s federal medicine regulator, the TGA, between mid-2022 and 2025, by medicinal cannabis product category. The study builds on a previous study that analysed voluntary adverse event reports for medicinal cannabis reported to the TGA between 2016 and early 2023 (Graham et al., 2023a).

## Methods

### Study design

This is a retrospective analysis of voluntary medicinal cannabis adverse event reports to the TGA between mid-2022 and 2025. Data were accessed from an existing Freedom of Information (FOI) log entry 26-1890 (Australian Government Department of Health, Disability and Ageing, 2025). As FOI data is deidentified and publicly available, the project was deemed exempt from the requirement for human research ethics approval.

### Inclusion and exclusion criteria

Unregistered medicinal cannabis products were included, with registered medicinal cannabis products (e.g. Sativex, Epidyolex) excluded from the analysis.

### MedDRA application

Adverse events were grouped by the Medical Dictionary for Regulatory Activities (MedDRA) terms used by regulators to code the adverse events reported (Medical Dictionary for Regulatory Activities (MedDRA), 2025). FOI adverse event data included MedDRA ‘Preferred Term’ (PT) level terminology (hereafter described as adverse events) for coding single medical concepts, including symptoms and diagnoses. A duplicated entry of oral paraesthesia was detected in one case report and was counted as one adverse event. Consensus decisions involving two researchers were used to assign the highest level of the MedDRA coding hierarchy, namely the ‘System Organ Classes’ (SOC) terminology (hereafter referred to as adverse event category), which can include aetiology, location and product-related issues. Product issues include device and product-related considerations, including quality.

### TGA medicinal cannabis categories and dosage forms

Medicinal cannabis product TGA categories were applied to the medicinal cannabis products by two researchers in duplicate (M.G., C.T.) using categories supplied by sponsors to the TGA from an additional FOI request 26-2535 (Australian Government Department of Health, Disability and Ageing, 2026), cannabis company product information and packaging images, and public TGA medicinal cannabis product category lists (Therapeutic Goods Administration, 2025a). Any differences in categorisation were resolved through discussion. Where there was insufficient information to determine TGA medicinal cannabis categories, the product was denoted as ‘uncategorised’. Reports involving multiple TGA medicinal cannabis product categories (*n* = 30) were coded as ‘mixed categories’. Dosage forms were included inconsistently, and dosage forms were determined from the product trade name and FOI 26-2535, where possible.

### Analysis

Descriptive analyses were conducted to report adverse event findings by MedDRA SOC and TGA composition categories. Analyses were conducted in STATA v18 and Excel.

## Results

Between 6 July 2022 and 31 May 2025, *n* = 615 cases and *n* = 1125 adverse events were reported across all medicinal cannabis categories. One report involving a registered product (e.g. Epidyolex) was excluded, leaving *n* = 614 cases and *n* = 1124 adverse events overall. The mean number of adverse events reported per case was 1.8 (SD = 1.5), with a range of 1–17. Reports were primarily from health professionals (*n* = 528/614; 86.0%), with fewer reports from patients/consumers (*n* = 70; 11.4%), pharmaceutical companies (*n* = 15; 2.4%) and ‘distributor or other organisation’ (*n* = 1; 0.2%). Across all TGA medicinal cannabis product categories, the top 3 MedDRA SOC were *Psychiatric disorders* (*n* = 344/1124; 30.6%), *Nervous System Disorders* (*n* = 201; 17.9%) and *Gastrointestinal disorders* (*n* = 161; 14.3%) (Table 1). Fourteen cases involved suicidal ideation (*n* = 9), suicidal behaviour (*n* = 4) or suicide attempt (*n* = 1).

**Table 1.** Top 10 MedDRA system organ class adverse event categories and top 3 preferred term adverse events for unregistered medicinal cannabis products.^a^

	System organ class	MedDRA^b^ preferred term	*n*	%
*1*	**Psychiatric disorders**		**344**	**30.6%**
		*Anxiety*	*76*	22.1%
		*Psychotic disorder*	*42*	12.2%
		*Paranoia*	*34*	9.9%
*2*	**Nervous system disorders**		**201**	**17.9%**
		*Headache*	65	32.3%
		*Somnolence*	*35*	17.4%
		*Dizziness*	20	10.0%
*3*	**Gastrointestinal disorders**		**161**	**14.3%**
		Nausea	57	35.4%
		Abdominal discomfort	27	16.8%
		Vomiting	24	14.9%
*4*	**Respiratory, thoracic and mediastinal disorders**		**117**	**10.4%**
		Cough	52	44.4%
		Dyspnoea	16	13.7%
		Oropharyngeal pain	11	9.4%
*5*	**Product issues**		**78**	**6.9%**
		Product complaint	22	28.2%
		Product quality issue	11	14.1%
		Product contamination microbial	8	10.3%
*6*	**General disorders and administration site conditions**		**68**	**6.0%**
		Drug ineffective	13	19.1%
		Condition aggravated	11	16.2%
		Malaise	7	10.3%
*7*	**Cardiac disorders**		**29**	**2.6%**
		Palpitations	13	44.8%
		Chest pain	5	17.2%
		Chest discomfort	4	13.8%
*8*	**Injury, poisoning and procedural complications**		**23**	**2.0%**
		Overdose	3	13.0%
		Product dispensing error	3	13.0%
		Product prescribing issue	3	13.0%
*9*	**Skin and subcutaneous tissue disorders**		**21**	**1.9%**
		Rash	6	28.6%
		Hyperhidrosis	4	19.0%
		Urticaria^c^	2	9.5%
*10*	**Metabolism and nutrition disorders**		**15**	**1.3%**
		Increased appetite	7	46.7%
		Decreased appetite	6	40.0%
		Appetite disorder	1	6.7%

a Case reports can include more than one adverse event. Preferred Terms are calculated as a proportion of each System Organ Class.

b Medical dictionary for regulatory activities.

c Equal third place with ‘rash pruritic’ and ‘lip swelling’.

Dried flower was the most common dosage form involved in cases (*n* = 284/614; 46.3%), followed by oral liquid (*n* = 167) and e-cigarette products (*n* = 44). Dosage form information was not reported or could not be determined for 64 reports (10.4%). Category 5 products were predominantly reported as dried flower (*n* = 270/332; 81.3%) or inhalation (*n* = 34; 10.2%) or either (*n* = 3). Category 1 (CBD > 98%) (*n* = 43/49; 87.8%) and Category 3 (balanced THC/CBD) (*n* = 49/67; 73.1%) were primarily oral liquid.

Half of all adverse event case reports were related to Category 5 medicinal cannabis products (*n* = 332/614; 54.1%). This was followed by Category 3 (balanced THC/CBD) products (*n* = 67/614; 10.9%) and Category 1 (CBD > 98%) products (*n* = 49/614; 8.0%). Across all product categories, most cases (*n* = 540/614; 87.9%) involved a single medicinal cannabis product (M = 1.16, SD = 0.51). Most cases relating to Category 5 products involved a single product (*n* = 279/332; 84.0%).

For Category 5 products, *Psychiatric disorders* were the leading adverse event category (*n* = 185/580; 31.9%), with the three most frequent adverse events being anxiety (*n* = 54), psychotic disorder (*n* = 18) and paranoia (*n* = 17). This is followed by *Nervous system disorders* (*n* = 112/580; 19.3%) and *Respiratory, thoracic and mediastinal disorders* (*n* = 84/580; 14.5%). *Gastrointestinal disorders* were the leading adverse event category for Category 1 (CBD > 98%) (*n* = 24/81; 29.6%) and Category 3 (balanced THC/CBD) products (*n* = 26/121; 21.5%).

## Discussion

**Figure 1.**
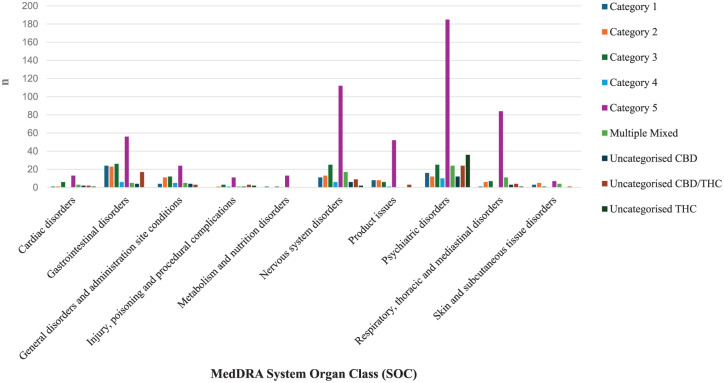
Top 10 MedDRA system organ classes by TGA medicine categorisation 2022–2025.

In all top 10 MedDRA adverse event categories, Category 5 products were most commonly implicated in voluntary adverse event reports (see Figure 1), coinciding with the increased prescription approvals for inhaled Category 5 products (Therapeutic Goods Administration, 2025b). *Psychiatric disorders* were the leading category of adverse events reported overall and for Category 5 products. The proportion of *Psychiatric disorders*, including anxiety, psychotic disorder and suicidal ideation/behaviours/attempt, raises concerns about whether patients are being effectively screened for risk factors and monitored.

These findings are in contrast to a previous analysis of TGA medicinal cannabis voluntary adverse events (29 September 2016 to 21 March 2023), where *Nervous System Disorders* were the leading category (Graham et al., 2023a). Moreover, previously, most cases involved combination CBD and THC products (*n* = 324/549; 59.0%) with few involving THC only products (*n* = 8/549; 1.5%). However, the analyses are not directly comparable due to the availability and redaction of different FOI cases and different data variables being available at the two timepoints. The results demonstrate a meaningful shift in the types of products associated with adverse events in recent years.

In parallel with increased prescription application approval trends for inhaled dosage forms, respiratory disorders are now ranked as the fourth adverse event category. The Category 5 e-cigarette and dried herb use as the primary exposure in this dataset suggests that, among those experiencing harm, current practice may not align with prescribing guidance (Graham et al., 2023b).

## Limitations

There are known limitations of adverse event reporting, including uncertainty around causality and duplicate reporting, and this report is subject to the same limitations (Graham et al., 2023a). It is possible that heightened media attention about Category 5 products increased selective reporting of related adverse events, or that increased use of higher strength products has driven increased harm. Some harms may have occurred in the context of nonmedical use, from diverted medicinal cannabis products or in the case of uncategorised medicinal cannabis products (*n* = 70) from products sourced outside of the legal market. This does not negate the importance of these findings, given that these complexities reflect real-world use and may indicate greater attention is needed to capture product sources as part of adverse event reporting. It is unknown what proportion of the cases involving dried herb pertains to vaporised versus smoked use. As concomitant medications and comorbidities were not reported, the extent drug- or disease-state interactions could have contributed to reported adverse events is unknown. Given that voluntary adverse event reports capture only a small proportion of adverse events, the rising frequency of these events is of concern, and the true prevalence of these harms is almost certainly higher.

## Conclusion

In the context of rapidly increasing prescription approvals for higher concentration THC medicinal cannabis products, we observed a predominance of psychiatric adverse events, including anxiety and psychotic disorders. In parallel with increased prescription approvals for inhaled products, respiratory adverse events rank fourth among the top 10 MedDRA categories reported. The high number of psychiatric disorders with medicinal products suggests that, in clinical practice, vulnerable people (i.e. those at highest risk of adverse events) are not being effectively screened and managed.
